# Alcohol Exposure Decreases CREB Binding Protein Expression and Histone Acetylation in the Developing Cerebellum

**DOI:** 10.1371/journal.pone.0019351

**Published:** 2011-05-31

**Authors:** Weixiang Guo, Erin L. Crossey, Li Zhang, Stefano Zucca, Olivia L. George, C. Fernando Valenzuela, Xinyu Zhao

**Affiliations:** Department of Neurosciences, University of New Mexico School of Medicine, Albuquerque, New Mexico, United States of America; National Institutes of Health, United States of America

## Abstract

**Background:**

Fetal alcohol exposure affects 1 in 100 children making it the leading cause of mental retardation in the US. It has long been known that alcohol affects cerebellum development and function. However, the underlying molecular mechanism is unclear.

**Methodology/Principal Findings:**

We demonstrate that CREB binding protein (CBP) is widely expressed in granule and Purkinje neurons of the developing cerebellar cortex of naïve rats. We also show that exposure to ethanol during the 3^rd^ trimester-equivalent of human pregnancy reduces CBP levels. CBP is a histone acetyltransferase, a component of the epigenetic mechanism controlling neuronal gene expression. We further demonstrate that the acetylation of both histone H3 and H4 is reduced in the cerebellum of ethanol- treated rats.

**Conclusions/Significance:**

These findings indicate that ethanol exposure decreases the expression and function of CBP in the developing cerebellum. This effect of ethanol may be responsible for the motor coordination deficits that characterize fetal alcohol spectrum disorders.

## Introduction

Fetal Alcohol Spectrum Disorder (FASD) is associated with persistent deficits in motor coordination and balance that are likely caused, in part, by alterations in the normal trajectory of cerebellar development [1], [2], [3]. Studies with rodents suggest that long-lasting cerebellar damage can occur after exposure during any stage of pregnancy, including the period equivalent to the human 3^rd^ trimester of pregnancy which corresponds to approximately the first 10–12 days of life in these animals [4]. Exposure to high (400 mg/dL = 87 mM) blood alcohol levels (BALs) during a portion of this period (postnatal days 4–7) was shown to significantly decrease Purkinje and granule cell numbers [5]. Electrophysiological alterations in the function of the Purkinje neurons that survive ethanol exposure during this period were demonstrated by Backman et al [6], who found a decrease in Purkinje neuron complex spike frequency that could be detected during adulthood. Moreover, exposure of rats to ethanol (330 mg/dL = 72 mM) during postnatal days 4–9 produced deficits in eye-blink conditioning, a behavioral paradigm that assesses the integrity of cerebellar-brain stem circuitry [4], [7]. Although the mechanisms responsible for these structural and functional cerebellar abnormalities are not fully understood, studies suggest that they involve alterations in the function of retinoic acid [8], growth factors [9], [10], [11], [12], ion channels [13], [14], [15], neurotransmitter systems [16], Ca^2+^ and cyclic nucleotide signaling pathways [17], cell cycle proteins [18], antioxidant protective mechanisms [19], [20] and cell adhesion molecules [21], [22]. The widespread effects of ethanol suggest that regulatory factors that impact multiple cellular pathways might be affected. However, such factors have not been identified.

A mechanism that could underlie the actions of ethanol on the developing cerebellum is disruption of epigenetic mechanisms mediated by DNA methylation, histone modification, and noncoding RNAs [23]. Epigenetic mechanisms can translate environmental influences into changes in the expression of genes that are known to have a significant role in brain development, as well as the pathophysiology of neurodevelopmental disorders [24], [25], [26], [27]. Importantly, studies suggest that DNA methylation and noncoding RNAs are altered in animal models of FASD [28]. For example, [29] observed evidence consistent with DNA hypomethylation and decreased nuclear methylase activity in samples isolated from whole day-11 embryos that were exposed to ethanol *in utero* during gestational days 9–11. [30] reported that exposure of whole mouse cultured embryos to a high concentration of ethanol (404 mg/dl = 88 mM) increased DNA methylation of genes located on chromosomes 7, 10, and X, including genes involved in growth, cell cycle and programmed cell death. [31] demonstrated that ethanol (322 mg/dl = 70 mM) exposure decreased expression of four noncoding microRNAs (miR-21, -335, -9, and -153) in cultured neurospheres from fetal mouse cerebral cortex, leading to cell cycle induction and stem cell maturation. However, whether alterations in histone modification play a role in the pathophysiology of FASD remains an open question.

The nucleosome is the fundamental unit of chromatin and is composed of DNA wrapped around an octameric protein core containing 4 different types of histones (H2A, H2B, H3 and H4) [24]. The N-terminal domains of these histones contain amino acid residues that can be enzymatically modified (for example, acetylated, phosphorylated, methylated, ubiquitinated and sumoylated) and this can have a powerful effect on chromatin structure[32]. In the nervous system, the best characterized histone modification is acetylation of N-terminal lysine residues, which typically shifts the conformation of chromatin into a relaxed state, leading to upregulation of gene transcription [33]. Histone acetylation levels are determined by the balance of the activities of two families of enzymes: histone acetyl transferases (HATs) and histone deacetylases (HDACs). At least four families of HATs have been identified based on sequence homology [34]. Among these, one of the best characterized is the CREB binding protein (CBP or CREBBP) family of HATs. CBP was originally identified as a co-activator for CREB and was later discovered to be a cofactor for many transcription factors, facilitating induction of the genes targeted by these factors. CBP can act both as a scaffolding protein for the transcription complex by recruiting components of the transcriptional machinery and via its intrinsic HAT activity, which ultimately causes chromatin relaxation. Therefore, CBP links classic gene regulatory mechanisms with epigenetic mechanisms. Both CREB and CBP are regulated by neuronal activity and their interaction is critical for activation of neuronal genes that are involved in brain development. Heterozygotic deficiency of CBP (and a related HAT, p300) causes the Rubinstein-Taybi Syndrome, a dominant mental retardation syndrome that is also characterized by growth deficits and distinct facial features[35]. Genetic mouse models with CBP mutations have been generated. Homozygote mutant mice are embryonically lethal, while heterozygote mice with about 50% reduction in CBP levels have impaired late-phase hippocampal long-term potentiation and learning deficits [36]. Deletion of CBP in the brain leads to reduced histone acetylation and learning deficits [37].

In addition to being crucial for normal hippocampal functioning, a number of studies suggest that CBP plays a central role in cerebellar development. First, cerebellar developmental abnormalities were observed in knockout mice for steroid receptor coactivator-1 (SRC-1), which possesses intrinsic HAT enzymatic activity as well as motifs that bind CBP/p300 and a number of nuclear receptors, including those for thyroid hormone, glucocorticoids, progesterone, estrogen and retinoic acid (reviewed in [38]). SRC-1 −/− mice display delayed Purkinje cell development and persistent motor alterations that could be a consequence of impaired responsiveness to steroid hormones [39]. Second, CBP expression and H4 acetylation have been shown to be reduced in a transgenic mouse model of spinocerebellar ataxia [40]. Finally, p300 has been shown to be a co-factor for RORα, an orphan nuclear receptor that is involved in Purkinje cell differentiation [41]. However, whether CBP is involved in ethanol-induced neurodevelopmental deficits is unknown. In light of these studies, we hypothesized that CBP might be a target of ethanol during cerebellar development. To test this hypothesis, we exposed rats to moderate levels of ethanol during the 3^rd^ trimester equivalent of human pregnancy and assessed the effect of ethanol exposure on CBP and acetylated histone levels in the developing cerebellum using western immunoblotting and immunohistochemical techniques. Our data unveil a novel target of ethanol in the developing brain and demonstrate a central role of epigenetic mechanisms in ethanol-induced neurodevelopmental deficits.

## Results

### CBP is expressed widely in neurons of developing cerebellum

**Figure 1:**
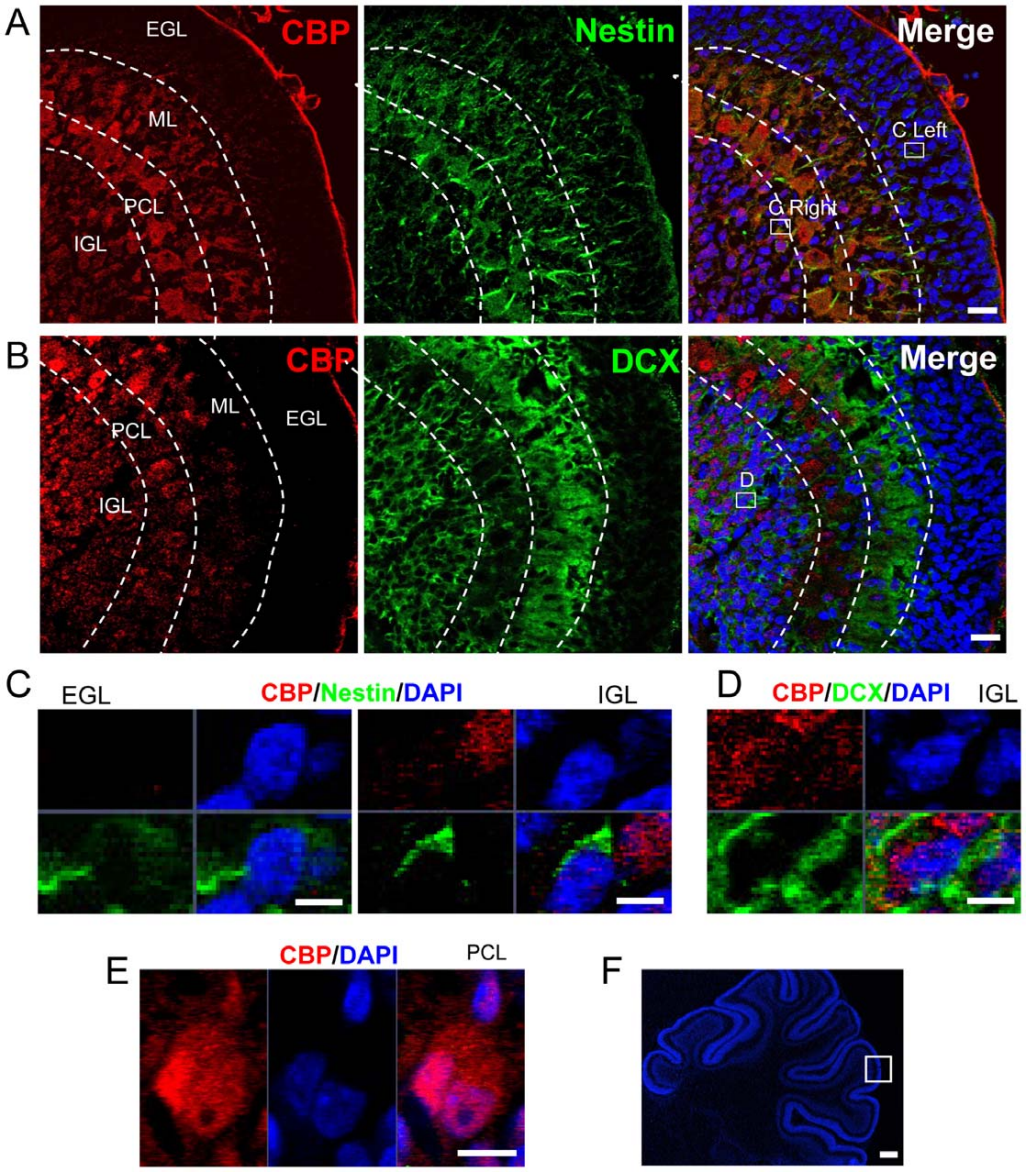
CBP expression is low in immature cells of the cerebellum at postnatal day 8. (A) CBP is nearly undetectable in Nestin+ ECL cells. Note that Nestin is expressed in the cells of the ML and PCL (see text for discussion). CBP, red; Nestin, green; Dapi, blue. EGL, external granular layer; ML, Molecular Layer; PCL, Purkinje cell layer; IGL, internal granular layer. Scale bar = 20 µm. (B) CBP is expressed at relatively low level in doublecortin (DCX+) young neurons in the ML. CBP, red; DCX, green; Dapi, blue. Scale bar = 20 µm. Boxes in A and B show the regions where magnification photos (C–E) are from. (C) High magnification confocal images showing that CBP expression is undetectable in Nestin+ EGL cells and very low in Nestin+ IGL cells. Note that in IGL, CBP is expressed at high level by a nearby Nestin-negative cell. Scale bar = 5 µm (D) High magnification confocal images showing that CBP is expressed by DCX+ cells in the IGL. Scale bar = 5 µm (E) High magnification confocal images showing that CBP is in both nucleus and cytosol of a Purkinje cell in the PCL. Scale bar = 10 µm. (F) A Dapi-stained cerebellar section showing the region for immunohistological analysis for Figures 1 and 2. Scale bar = 100 µm.

**Figure 2:**
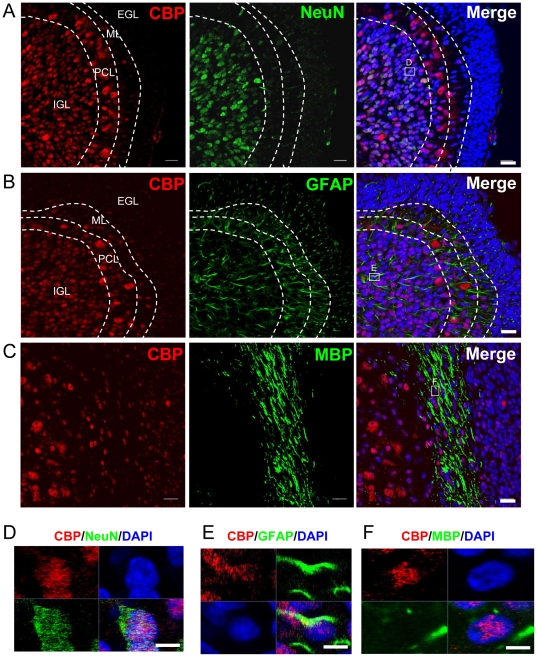
CBP is predominantly expressed in relatively mature neurons of the cerebellum at postnatal day 8. (A) CBP is expressed at high level in the nucleiof NeuN+ mature neurons in IGL. CBP is also expressed in Purkinje Cells of PCL and these cells do not express NeuN. CBP, red; NeuN, green; Dapi, blue. EGL, external granular layer; ML, Molecular Layer; PCL, Purkinje cell layer; IGL, internal granular layer. Scale bar = 20 µm (B) CBP is also expressed in a subset of astrocytes in ML, PCL, and IGL. Scale bar = 20 µm (C) CBP is also expressed in nuclei located in the MBP+ myelin track, suggesting that at least a subset of oligodendrocytes also expresses CBP. Scale bar = 20 µm (D) High magnification confocal images showing that CBP expression is colocalized with NeuN. Scale bar = 5 µm (E) High magnification confocal images showing that CBP is expressed by some GFAP+ cells. Scale bar = 5 µm (F) High magnification confocal images showing that CBP is in the nuclei located in the MBP+ myelin track. Scale bar = 5 µm.

To determine whether CBP is involved in cerebellar development, we first investigated which neuronal populations express CBP in the developing cerebellum from postnatal day 8 (P8) rats, by using immunohistochemical studies. At this age, the cerebellar cortex consists of four layers: external granule layer (EGL), molecular layer (ML), Purkinje cell layer (PCL), and the internal granule layer (IGL) [42], [43]. The EGL is mainly composed of immature proliferating granule cells. The ML contains Purkinje cell dendrites and migrating molecular layer interneurons. The Purkinje layer contains the cell bodies of these neurons, which are arranged in multiple layers at this developmental stage. The IGL consists of more mature granule cells, Golgi cells and mossy fibers. At P8, immature granule cells in the EGL are migrating (passing through ML and PCL) to become more mature granule neurons at IGL. As shown in Figure 1, CBP expression was nearly undetectable in the nestin+ EGL cells (Fig. 1A, 1C, and 1F), and low in doublecortin-positive (DCX+) ML neurons (Fig. 1B, 1D, and 1F), but high in PCL and IGL neurons (Fig. 1A, 1B, and 1F). We found that the young Purkinje cells also express nestin (Fig. 1), a finding that is consistent with the literature[44] and the Allan Brain Atlas (http://www.brain-map.org/). Interestingly, while CBP is located in granule neuron nuclei at IGL, it is both in the nucleus and cysotol of Purkinje cells in PCL (Fig. 1E). Using a mature neuronal marker, NeuN, we found that CBP was predominantly expressed in relatively more mature, non-migrating neurons, located in the IGL and PCL (Fig. 2A and D). A subpopulation of GFAP astroctyes also expressed CBP (Fig. 2B and E). In addition, CBP expression was detected in MBP+ myelin fibers (Fig. 2C and F), suggesting its expression in oligodendrocytes. In summary, CBP is expressed at high levels in neurons and also glial cells of the developing cerebellar cortex.

### Perinatal ethanol exposure leads to reduced CBP expression in the developing cerebellum

**Figure 3:**
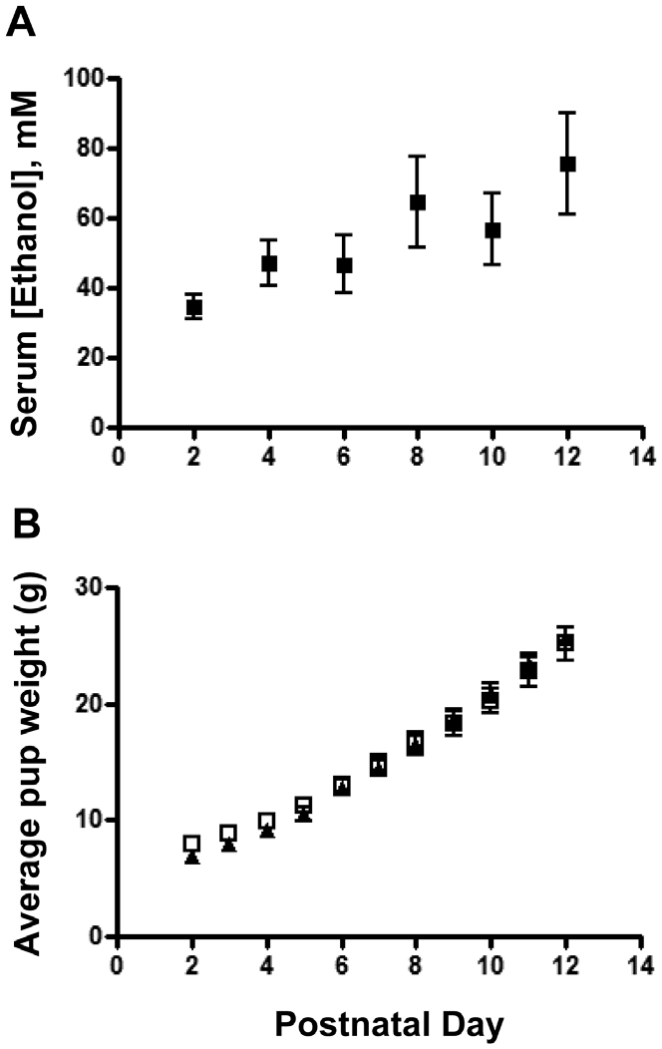
Moderate perinatal ethanol exposure does not affect pup weight. (A) Pup serum alcohol during the course of ethanol exposure between postnatal day 2 and day 12 (n = 6). (B) Ethanol exposure did not affect the weight of pups as assessed during postnatal day 2 to day 12. Control, solid triangles; ethanol, open squares; n = 9 litters per group.

**Figure 4:**
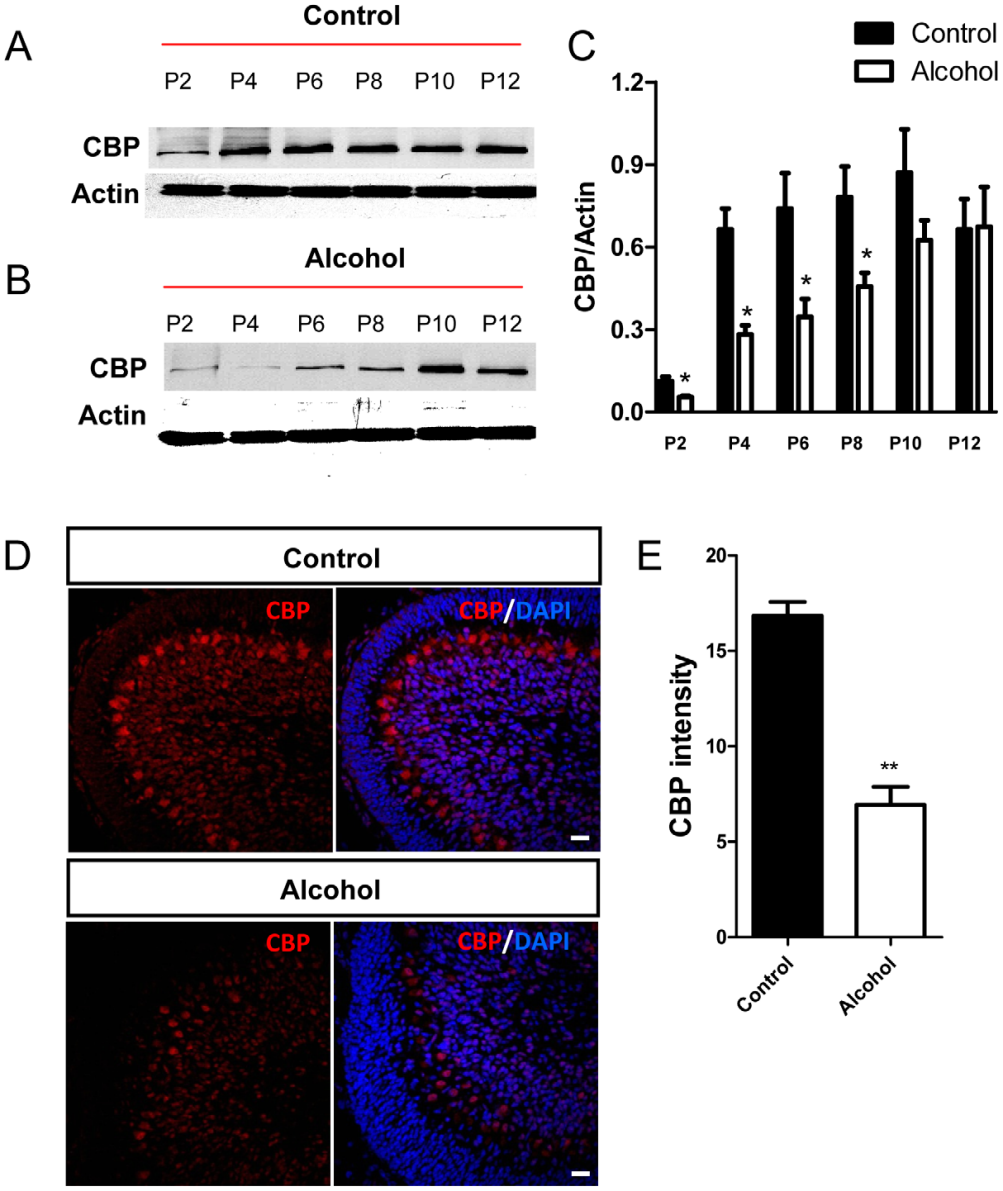
CBP protein expression was decreased in the cerebellum from ethanol-exposed developing rats. (A) Image of one of the western blots showing differential CBP expression in cerebellum isolated from control pups (exposed to air) analyzed at P2 to P12. (B) Representative CBP western blot analysis of cerebellum isolated from ethanol-exposed pups analyzed at P2 to P12. (C) Quantitative analysis showing that CBP protein levels (normalized to β-actin) are lower in the ethanol group compared to the control group between P2 and P10, but not at P12. Three independently exposed liters were analyzed and each was counted as n = 1. Multiple western blots were analyzed for each exposure to minimize the variation between western blots. (D) Representative CBP immunohisological images of control and ethanol exposed brains at P8. CBP, red;’ Dapi, blue Scale bar = 50 µm (E) Quantitative analysis of CBP immunohistological signal intensity demonstrates that CBP expression is reduced in ethanol exposed cerebellum compared to controls. N = 3 individual animal for P2 and P4/condition. N = 4 individual animal for P6 –P12/condition. *, P<0.05, t-test.

We next tested the effect of ethanol exposure on cerebellar CBP expression. Exposure to ethanol vapor resulted in serum ethanol concentrations in the pups that were similar across postnatal days (overall average = 54±4 mM; n = 48; Fig. 3A; for comparison, 80 mg/dl = 17.4 mM). Furthermore, pup weight gain was similar in the air and ethanol groups (Fig. 3B). There was only one case of neonatal mortality and it occurred in a litter exposed to air. In cerebellar homogenates from air-exposed animals, CBP expression increased approximately 4-fold between postnatal day 2 and postnatal day 4, and then remained relatively stable until postnatal day 12, the last day of our analysis (Fig. 4A, C). In cerebellar homogenates from ethanol-exposed animals, CBP levels were significantly lower compared to control pups between postnatal days 2 and 10 (Fig. 4B, C). Although both control and ethanol groups had similar CBP expression levels at postnatal day 12 (Fig. 4A–C), the developmental stage-dependent upregulation of CBP was much delayed in ethanol-exposed animals compared to control animals. Immunohistochemical experiments also detected a decrease in CBP levels in IGC and PCL at postnatal day 8 (Fig. 4D, E). Therefore, 3^rd^ trimester equivalent ethanol exposure led to reduced CBP expression in the developing cerebellum.

### Perinatal ethanol exposure leads to reduced histone acetylation in the developing cerebellum

**Figure 5:**
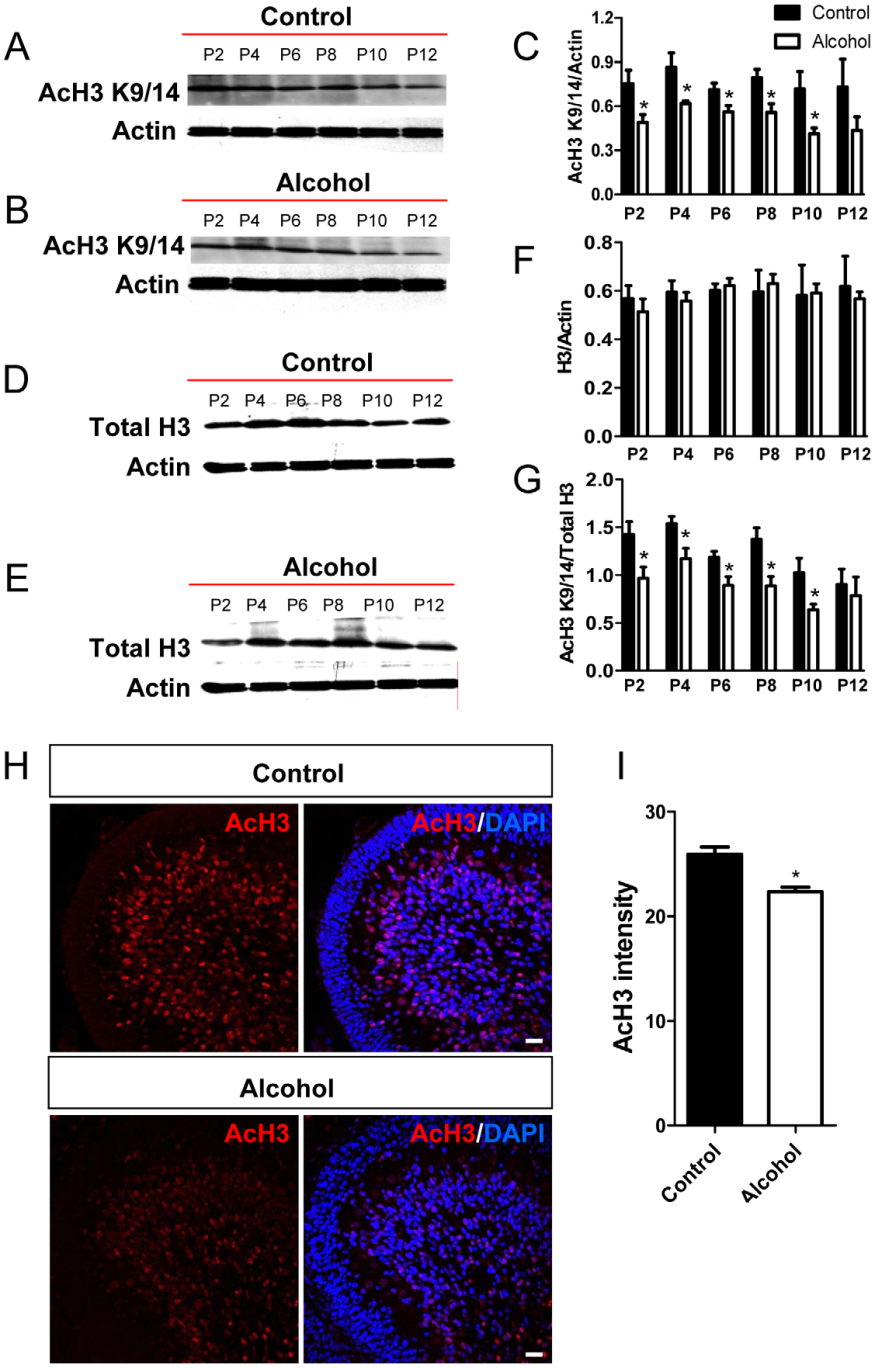
AcH3 expression was decreased in the cerebellum of ethanol-exposed developing rats. (A) Image of one of the western blots showing differential acetylated H3 (AcH3) expression in the cerebellum isolated from control pups (exposed to air) analyzed at P2 to P12. (B) Representative AcH3 western blot analysis of cerebellum isolated from ethanol-exposed pups analyzed at P2 to P12. (C) Quantitative analysis showing that AcH3 protein levels (normalized to β-actin) are lower in the ethanol group compared to the control group between P2 and P10, but not at P12. Three independently exposed liters were analyzed and each was counted as an n = 1. Multiple western blots were analyzed for each exposure to minimize the variation between western blots. (D) Representative total H3 (Total H3) western blot analysis of cerebellum isolated from control pups (exposed to air) analyzed at P2 to P12. (E) Representative total H3 western blot analysis of cerebellum isolated from ethanol-exposed pups analyzed at P2 to P12. (F) Quantitative analysis showing that total H3 protein levels (normalized to β-actin) are not changed in the ethanol group compared to the control group. (G) Quantitative analysis showing that the ratio of AcH3 compared to total H3 protein levels are lower in the ethanol group compared to the control group between P2 and P10, but not at P12. (H) Representative AcH3 immunohistological images of control and ethanol exposed cerebella at P8. AcH3, red; Dapi, blue. Scale bar = 50 µm. (I) Quantitative analysis of AcH3 immunohistological signal intensity demonstrates that AcH3 expression is reduced in the cerebellum of ethanol exposed rats compared to controls. N = 4 individual animal/condition. P<0.05 t-test.

Since CBP functions through its intrinsic HAT activity, we examined whether the decrease in CBP expression was associated with a reduction in histone acetylation levels. We chose to study histones type-3 (H3) and -4 (H4), as these are well known substrates of the HAT activity of CBP [37], [45] and CBP and AcH3 and AcH4 are colocalized in the developing cerebellum (Fig. S1). As shown in Fig. 5, in control brains, acetylated H3 levels were high at postnatal days 2–8 and decreased slightly during postnatal days 10–12 (Fig. 5A, C) while total H3 levels were constant (Fig. 5D, F) during this developmental period. Ethanol exposure significantly reduced acetylated H3 levels at postnatal days 2–10 (Fig. 5B, C), but had no significant effect on total H3 protein levels (Fig. 5E, F). Consequently, the levels of acetylated H3 normalized to total H3 protein were significantly reduced as a result of ethanol exposure from P2 through P10 (Fig. 5G). In addition, immunohistochemical studies revealed a decrease in acetylated H3 levels in cerebellar sections from the ethanol-exposed group at postnatal day 8 (Fig. 5H, I). To determine if the effect of ethanol exposure is specific for H3K9/K14 acetylation, we then used an antibody that specifically recognizes H3 acetylation at a different residue (K23) and found that the levels of acetyl-H3K23 were also reduced in alcohol-exposed group (Fig. S2).

**Figure 6:**
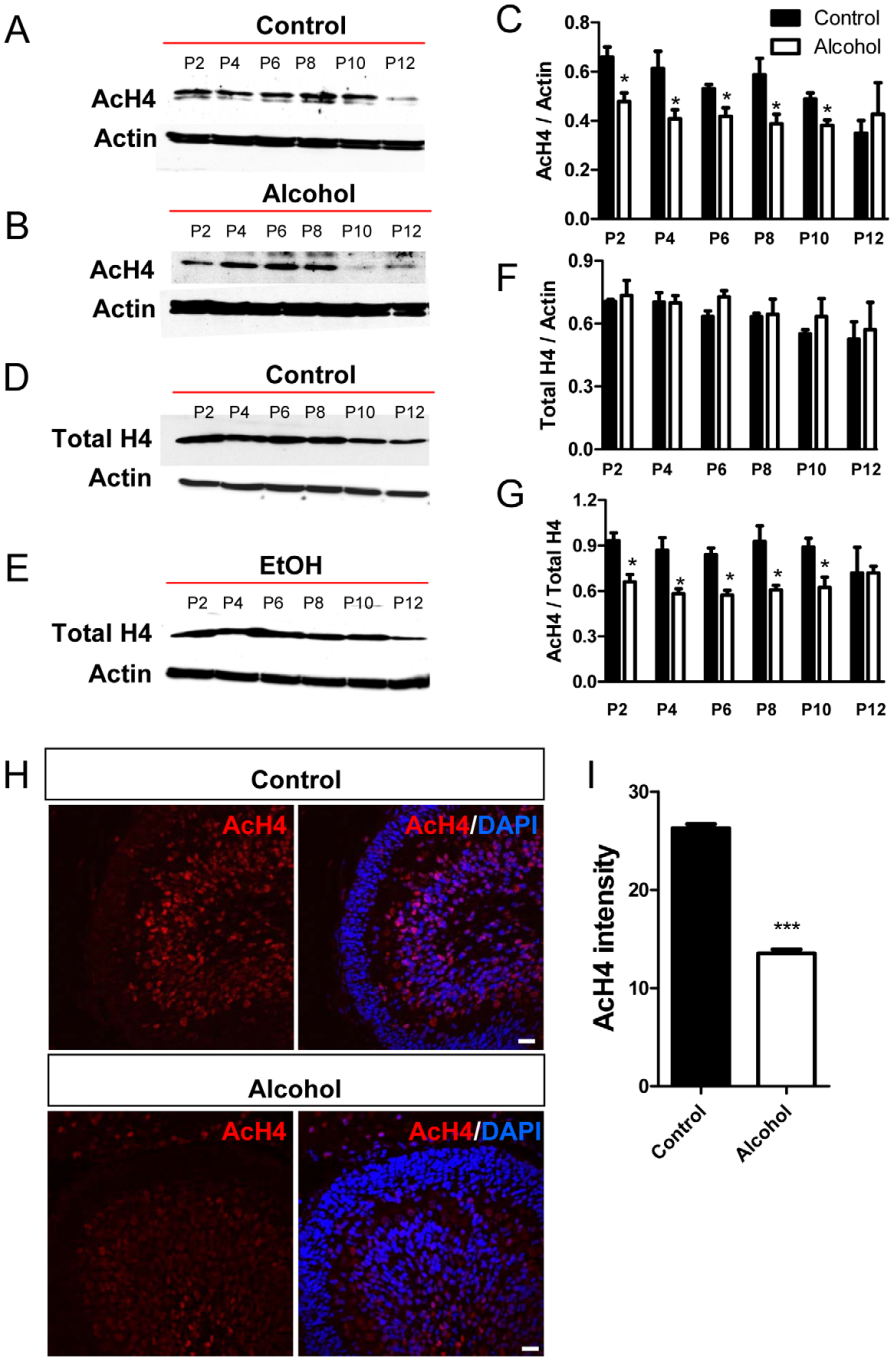
AcH4 expression was decreased in the cerebellum of ethanol-exposed developing rats. (A) Image of one of the western blots showing differential acetylated H4 (AcH4) expression in the cerebellum isolated from control pups (exposed to air) analyzed at P2 to P12. (B) Representative AcH4 western blot analysis of cerebellum isolated from ethanol-exposed pups analyzed at p2 to p12. (C) Quantitative analysis showing that AcH4 protein levels (normalized to b-actin) are lower in the ethanol group compared to the control group between P2 and P10, but not at P12. Three independently exposed liters were analyzed and each was counted as n = 1. Multiple western blots were analyzed for each exposure to minimize the variation between western blots. (D) Representative total H4 (Total H4) western blot analysis of cerebellum isolated from control pups (exposed to air) analyzed at P2 to P12. (E) Representative Total H4 western blot analysis of cerebellum isolated from ethanol-exposed pups analyzed at P2 to P12. (F) Quantitative analysis showing that total H4 protein levels (normalized to b-actin) are not changed in the ethanol group compared to the control group. (G) Quantitative analysis showing that the ratio of AcH4 compared to total H4 protein levels are lower in the ethanol group compared to the control group between P2 and P10, but not at P12. (H) Representative AcH4 immunohistological images of control and ethanol-exposed cerebella at P8. AcH4, red; Dapi, blue. Scale bar = 50 µm. (I) Quantitative analysis of AcH4 immunohistological signal intensity demonstrates that AcH4 expression is reduced in the cerebellum of ethanol exposed rats compared to controls. N = 4 individual animal/condition. P<0.05 t-test.

We next analyzed the acetylation of H4 (K5, 8, 12, and 16). In naïve animals, acetylated H4 levels were high at postnatal day 2 and decreased gradually until day 12 (Fig. 6A, C), and unlike total H3, total H4 protein levels also exhibited this gradual decrease (Fig. 6D, F). Similarly, ethanol exposure significantly reduced acetylated H4 levels compared to controls during postnatal days 2–10 (Fig. 6B, C) without having a significant effect on total H4 levels (Fig. 6E, F). Consequently, the levels of acetylated H4 normalized to total H4 protein were significantly reduced as a result of ethanol exposure from P2 though P10 (Fig. 6G). Immunohistochemical studies also revealed a decrease in acetylated H4 levels in cerebellar sections from the ethanol-exposed group at postnatal day 8 (Fig. 6H, I). Therefore, 3^rd^ trimester equivalent ethanol exposure led to reduced histone H3 and H4 acetylation, without affecting total histone H3 and H4 protein expression in the developing cerebellum.

## Discussion

In this study, we demonstrate that CBP is highly expressed in developing cerebellar neurons and ethanol exposure leads to nearly a 50% reduction of CBP levels as well as a reduction in histone acetylation. Given the important functions of CBP in many cellular pathways and the fact that heterozygote *CBP* gene mutation with 50% reduction of CBP protein levels results in human mental retardation, our discovery unveils a novel target of ethanol which may shed light on understanding the molecular mechanism underlying alcohol-induced neurodevelopmental deficits.

CBP is expressed in the forebrain of both developing and adult rodents [46]–[47] and CBP expression has been shown in the adult rat cerebellum [47]. In addition, CBP is also expressed in cultured cerebellar granule neurons isolated from developing brains [48], [49]. Furthermore, *in situ* hybridization data suggest that CBP mRNA is expressed in the developing cerebellum (Allan Brain Atlas). However, the presence of CBP protein and cell type-specific expression patterns of CBP in developing cerebellum have not been reported. Here, we show that CBP is predominantly expressed in neurons and, in particular, relatively mature neurons in the PCL and IGL. In addition, CBP expression increases as development progresses, with a particular sharp increase at P2–4, suggesting a critical role of this protein in neuronal maturation during cerebellar development. Although CBP expression patterns spatially match that of acetylated histones in the P8 brains, H3 and H4 acetylation, however, does not sharply increase from P2–4, suggesting that either other acetyltransferases might be more dominant at P2, or stronger HDAC activity might be present between P2–4. This is consistent with the fact that while ethanol leads to a nearly 50% reduction of CBP levels, it only reduces H3 and H4 acetylation levels by 20%. In addition, a reduction in CBP expression may lead to decreased acetyl histone binding to specific gene loci in addition to the global changes of histone acetylation. Future chromatin immunoprecipitation studies coupled to genome wide analysis will further unveil the impact of CBP reduction on specific gene expression and function in FASD models.

We found that ethanol exposure decreased CBP and acetylated levels for H3 and H4 in the developing cerebellum. These effects could be detected even after a single 4 hr ethanol exposure episode at P2. At P12, CBP and histone acetylation levels were not significantly different between the control and ethanol groups, consistent with the literature indicating that the developing cerebellum is particularly vulnerable to the actions of ethanol during the first 10 days of life [4]. During this period, rapid growth of axons and dendrites takes place in granule cells; Purkinje cells, Golgi cells and molecular layer interneurons, and synapses among these neurons are actively generated and refined. It will be interesting to directly establish whether these processes are, in part, mediated by CBP-dependent modulation of changes in gene expression. The decrease in CBP expression should impair gene expression driven by CREB; this effect may be enhanced by a parallel inhibitory effect of ethanol on phosphorylated CREB levels caused by dysregulation of [Ca^2+^]_i_, as demonstrated by Gruol et al in acutely dissociated Purkinje neurons from neonatal rats [50]. In developing Purkinje neurons, it is well established that progesterone and estradiol promote dendritic growth, generation of spines and synaptogenesis and these effects may be mediated by brain-derived neurotrophic factor (reviewed in [51]). The thyroid hormone is also important for these processes and is also essential for granule cell maturation and for myelination of cerebellar axonal tracts (reviewed in [52]). Glucocorticoids inhibit granule cell division and proliferation in the external granule cell layer, leading to a reduction in granule cell numbers in the internal granule layer [53]. Exposure to high levels of retinoic acid leads to the loss of a subpopulation of proliferating granule cells [54]. As mentioned above, SRC-1 is an important cofactor for the actions of these agents and CBP/p300 are required for the normal functioning of SRC-1 (reviewed in [38]). Therefore, it is possible that ethanol exposure during the 3^rd^ trimester equivalent alters cerebellar cortical development by blunting the trophic actions of the above-mentioned agents via a disruption in the facilitator actions of CBP/SRC-1.

To the best of our knowledge, this is the first study of the effect of ethanol exposure on CBP and histone acetylation levels in the developing brain. The effects of acute and chronic ethanol exposure on CBP and histone acetylation levels were previously characterized in male adult rats by Pandey et al[55]. These investigators found that acute administration of an anxiolytic dose of ethanol (1 g/kg intraperitoneally; BAL = 92 mg/dl) decreased HDAC activity and increased levels of CBP, and acetylated H3/H4 in the central and medial nuclei of the amygdala. In rats chronically treated with an ethanol-containing liquid diet for 15–16 days (BAL = 177 mg/dl) and then withdrawn for 24 hr, HDAC activity increased and levels of acetylated H3/H4, and CBP decreased. These findings are in general agreement with those of the present study, suggesting that CBP is an important target of ethanol both in developing and mature animals. Moreover, these findings support the possibility that ethanol affects CBP expression in multiple brain regions and this should be assessed in the future. Since a 50% reduction of CBP levels results in human Rubinstein-Taybi Syndrome, a dominant disorder that shares mental retardation, growth deficits, and certain facial features with fetal alcohol syndrome[35], our findings may shed light on a common pathway between genetic-based and toxin-induced mental retardation disorders.

The mechanisms responsible for the ethanol-induced decreases in CBP expression remain to be elucidated. Although pathways that regulate CBP activity (for instance, phosphorylation pathways) have been identified [56], [57], [58], the literature on factors that control expression of CBP is scarce. Interestingly, it was demonstrated that activation of apoptotic pathways triggered degradation of CBP and histone deacetylation in cultured cerebellar granule neurons from postnatal day 7 mice that were maintained in culture for 6 days. CBP degradation was mediated by caspase-6 and calpain [48]. Exposure to high doses of ethanol (∼300–500 mg/dl for at least 8 hr) during the 3^rd^ trimester-equivalent has been shown to activate apoptotic pathways in the developing rat cerebellum [59], [60]. Calpain activation has also been demonstrated in the cerebral cortex in rats exposed to high doses of ethanol during postnatal days 4–10 [61]. Therefore, it is important to determine whether ethanol induces degradation of CBP via a caspase/calpain-dependent mechanism and if this can occur at moderate doses of ethanol or can only be observed in response to high dose ethanol exposure. It should be noted that ethanol exposure decreases CBP mRNA expression (Zhao and Valenzuela, unpublished observation), suggesting that ethanol could act at the transcriptional level and we are currently actively investigating this possibility.

In conclusion, our findings demonstrate that ethanol exposure during the 3^rd^ trimester-equivalent of human pregnancy decreases CBP and acetylated histone levels in the developing cerebellum. Future studies should determine whether this effect can occur in other regions of the brain and other organ systems. It should also be investigated if exposure during other stages of pregnancy can also reduce CBP expression. Ongoing studies in our laboratories are exploring the impact of these effects of ethanol on expression of genes that are controlled by this key transcriptional regulator/integrator. In light of the findings of Pandey et al[55] indicating that HDAC inhibitors could be useful in the treatment of alcohol withdrawal-induced anxiety, it is also important to investigate the potential value of these agents in the treatment of FASD.

## Methods

### Ethics Statement

Animal procedures were approved by the Institutional Animal Care and Use Committee of the University of New Mexico Health Sciences Center and conformed to National Institutes of Health guidelines. The Animal Protocol approved for this study is #08UNM012 (PI, C. Fernando Valenzuela).

### Exposure of neonatal rats to ethanol vapor and preparation of brain samples

Animal procedures were approved by the Institutional Animal Care and Use Committee of the University of New Mexico Health Sciences Center and conformed to National Institutes of Health guidelines. Timed-pregnant Long-Evans rats were ordered from Harlan (Indianapolis, IN) and housed individually with *ad libitum* access to chow and water under a 12 hr light:dark cycle (lights on at 6 am). Male and female neonatal rat pups and their respective mothers were exposed to ethanol for 4 hr/day between postnatal days 2 to 12 in inhalation chambers (La Jolla Research Inc, La Jolla, CA), as previously described [62]. Litters were culled to 8–12 pups and exposed to air or air plus ethanol. This paradigm models repeated daily maternal drinking sessions during the third trimester-equivalent of human pregnancy. An advantage of this paradigm is that it produces low blood ethanol levels in the dams and does not disrupt grooming and nursing behavior [62]. This paradigm produced serum ethanol concentrations that are approximately twice the legal intoxication limit and does not affect pup weight gain (Fig. 3).

Within 1 hr of the end of the 4 hr ethanol exposure, pups were euthanized by decapitation under ketamine (250 mg/kg) anesthesia and trunk blood collected. The concentrations of ethanol in pup serum were then determined using a standard alcohol dehydrogenase-based assay [62]. For Western blotting studies, cerebella were dissected, flash frozen in liquid nitrogen and stored at -80°C. For immunohistochemical studies, cerebella were fixed in 4% paraformaldehyde for 24 hours at 4°C, rinsed in phosphate-buffered saline (PBS; pH 7.4) and then cryoprotected in 30% sucrose (wt/v) in PBS for 48 hours. Brains were quickly frozen in Optimal Cutting Temperature reagent (Sakura Finetek, Torrance, CA) and coronal sections (14–18 µm) were collected on Superfrost-plus slides. Sections were air dried at room temperature and stored at −80°C.

### Western blot

Western blotting analyses were performed as previously described[63], [64]. Cerebellar tissues were dissected from postnatal rat pups and frozen immediately in liquid nitrogen until use. Tissues were homogenized in RIPA buffer (50 mM Tris HCl pH 8, 150 mM NaCl, 1% NP-40, 0.5% sodium deoxycholate, 0.1% SDS) or RIPA buffer with 2% SDS (for Figure S1) and total protein concentration was measured by using Bio-Rad protein assay kit (#500-0006; Bio-Rad, Hercules, CA). Twenty-µg of each protein sample was separated on SDS-PAGE gels and transferred to PVDF membranes (Millipore, Billerica, MA). After primary and secondary antibody incubation, specific protein signals were visualized by processing membranes using enhanced chemiluminescence (GE Healthcare, Piscataway, NJ). The following primary antibodies were used at the concentrations recommended by the manufacturers: rabbit anti-CBP (1∶200, #SC-369, Santa Cruz Biotechnology, Santa Cruz, CA), rabbit anti-Histone H3 (1∶1000, #06-755, Millipore, Billerica, MA), rabbit anti-Histone H4 (1∶1000, #07-108, Millipore, Billerica, MA), rabbit anti-acetyl-Histone H3 (1∶1000, #06-599, Millipore, Billerica, MA), rabbit anti-acetyl-Histone H3 (K23) (1∶1000, #9674, Cell Signaling, Danvers, MA), rabbit anti-acetyl-Histone H4 (1∶1000, #06-866, Millipore, Billerica, MA), and mouse anti-β-Actin (1∶1000, #A2228, Sigma-Aldrich, St. Louis, MO). HRP-conjugated secondary antibodies were purchased from Thermo Fisher Scientific (Rockford, IL). For loading controls, membranes were stripped and reprobed with the antibody against β-Actin. Chemiluminescence was captured on X-ray films which were scanned and the intensities of the bands of interest were quantified via ImageJ software version 1.43 (NIH). The relative quantity of the proteins of interest were obtained by normalizing to the expression levels of β-Actin or, in some cases, total histone H3 or H4 levels.

### Immunohistology and quantification

Immunohistological staining and confocal imagings were performed as previously described[63], [64]. Briefly, frozen brain sections were stained with following primary antibodies: rabbit anti-CBP (1∶200, #SC-369, Santa Cruz Biotechnology, Santa Cruz, CA), mouse anti-CBP (1∶100, #LS-C88255, Lifespan Bioscience, Seattle WA), mouse anti-NeuN (1∶1000, #MAB377, Millipore, Billerica, MA), mouse anti-MBP (1∶1000, Millipore, 05-675, Billerica, MA), chicken anti-Nestin (1∶1000, #mNES, Aves Lab, Inc, Tigard, Oregon), goat anti-Doublecortin (1∶200, #SC-8066, Santa Cruz, Santa Cruz, CA), anti-acetyl-Histone H3 (1∶1000, #06-599, Millipore, Billerica, MA), rabbit anti-acetyl-Histone H4 (1∶1000, #06-866, Millipore, Billerica, MA). Fluorescent secondary antibodies were used at 1∶250 dilutions (donkey from Jackson ImmunoResearch, West Grove, PA or goat from Invitrogen, Carlsbad, CA). After staining, sections were mounted, coverslipped, and maintained at 4°C in the dark until analysis. Confocal images were acquired at 40× by using LSM 510 Image Examiner (Carl Zeiss, Thornwood, NY) and imported into ImageJ. Signal intensities of AcH3 and AcH4 were quantified using ImageJ software version 1.43 (NIH). Ten individual cells were picked randomly from brain sections of each animal and measured for signal intensities. The average intensity from each animal (10 cells) was count as n = 1 for statistics. Samples from three individual animals each from a different litter per experimental condition were analyzed (n = 3).

### Statistical Analysis

Unless specified, statistical analysis was performed using ANOVA and Student t-test, with the aid of SPSS v.17. All data are shown as mean with standard error of mean (mean±SEM). p<0.05 was considered significant.

## Supporting Information

Figure S1 **Colocalization of CBP and acetylated histones in the developing cerebellum.** (A, B) Confocal images showing collocalization of CBP and AcH3 in the developing cerebellum. AcH3 (green), CBP (red), DAPI (blue). (C, D) Confocal images showing collocalization of CBP and AcH3 in the developing cerebellum. AcH3 (green), CBP (red), DAPI (blue). Scale bar in A and C = 20 µm; scale bar in B and D = 10 µm.
(PDF)

Figure S2 **AcH3K23 expression was decreased in the cerebellum of ethanol-exposed developing rats.** (A) Image of one of the western blots showing acetylated H3K23 (AcH3K23) expression in the cerebellum isolated from control pups (exposed to air) analyzed at p2 to p12. (B) Image of one of the western blots showing AcH3 expression in the cerebellum isolated from ethanol-exposed pups analyzed at p2 to p12. (C) Quantitative analysis showing that AcH3 protein levels (normalized to β-actin) are lower in the ethanol group compared to the control group between P2 and P10, but not at P12. (D) Quantitative analysis showing that total H3 protein levels (normalized to β-actin) are not changed in the ethanol group compared to the control group. (E) Quantitative analysis showing that the ratio of AcH3K23 compared to total H3 protein levels are lower in the ethanol group compared to the control group between P2 and P12. Three independently exposed liters (n = 3) were analyzed and each was counted as one replicate. Multiple western blots were analyzed for each exposure to minimize the variation between western blots. For P2 and P10, only two independent liters were analyzed therefore no statistic analysis was done on the data for these two time points.
(PDF)
