# Supplementary figures and images for: Alcohol Exposure Decreases CREB Binding Protein Expression and Histone Acetylation in the Developing Cerebellum
Source: PLoS One. 2011 May 31;6(5):e19351. doi: 10.1371/journal.pone.0019351 (PMC3104983; doi:10.1371/journal.pone.0019351)

Supplemental Figure S1

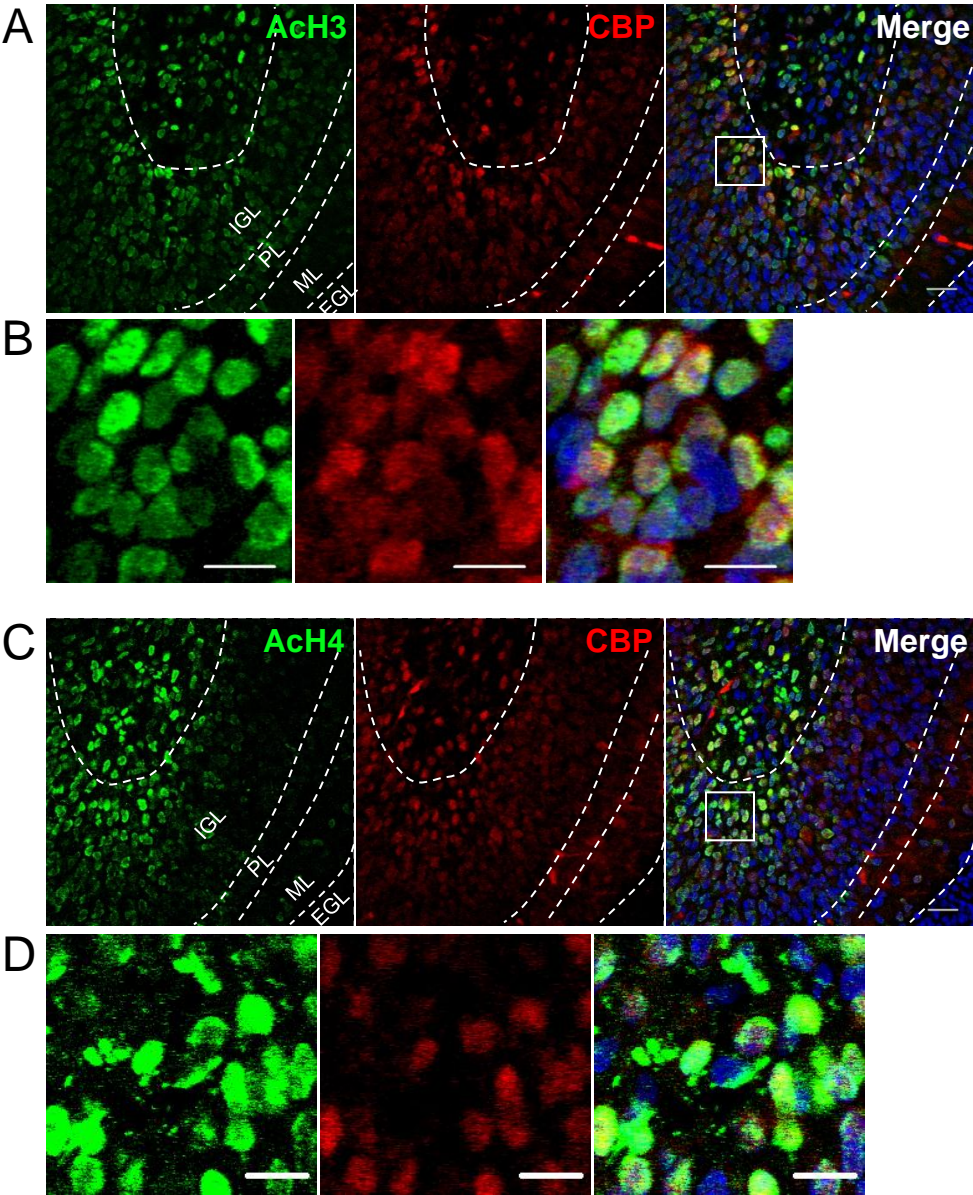

Supplement: Figure S1 — Colocalization of CBP and acetylated histones in the developing cerebellum. (A, B) Confocal images showing collocalization of CBP and AcH3 in the developing cerebellum. AcH3 (green), CBP (red), DAPI (blue). (C, D) Confocal images showing collocalization of CBP and AcH3 in the developing cerebellum. AcH3 (green), CBP (red), DAPI (blue). Scale bar in A and C = 20 µm; scale bar in B and D = 10 µm. (PDF) [file pone.0019351.s001.pdf]

Supplemental Figure S2

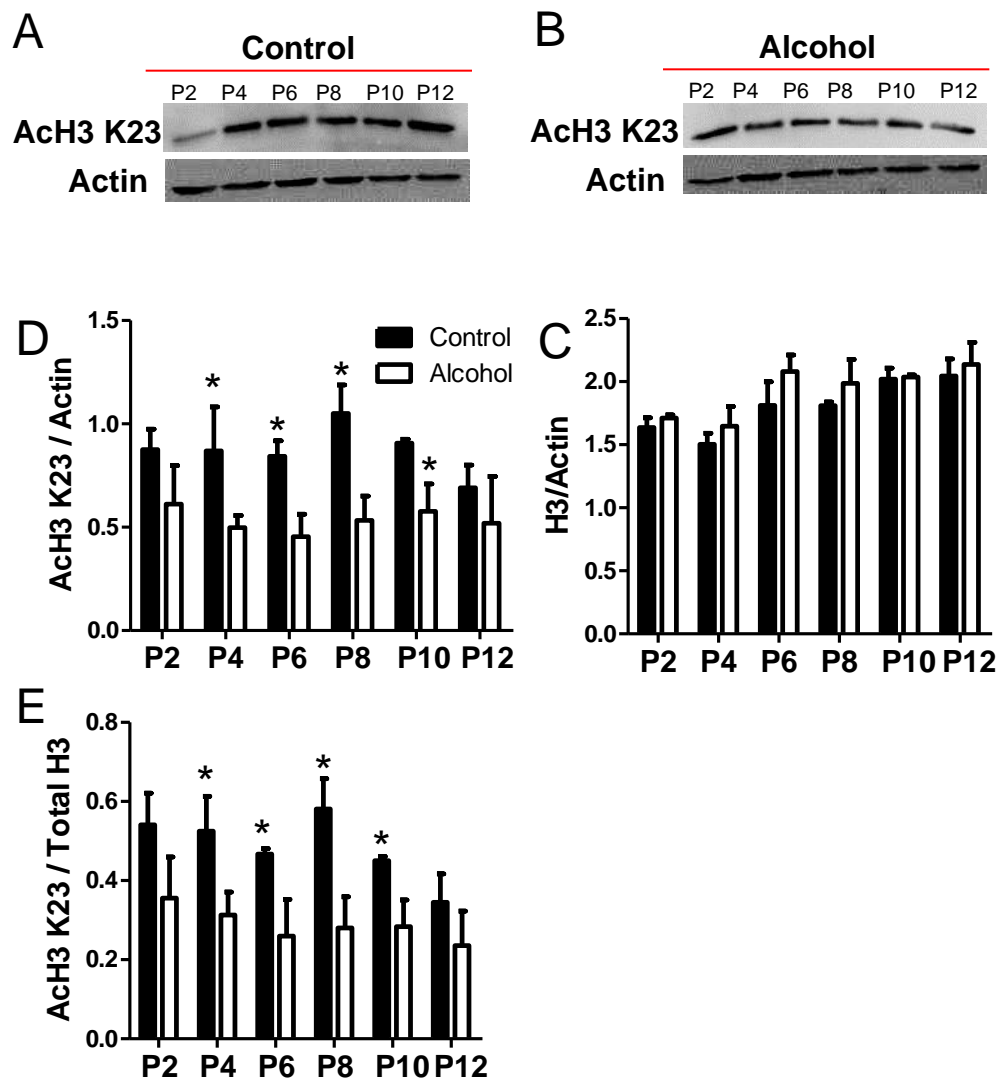

Supplement: Figure S2 — AcH3K23 expression was decreased in the cerebellum of ethanol-exposed developing rats. (A) Image of one of the western blots showing acetylated H3K23 (AcH3K23) expression in the cerebellum isolated from control pups (exposed to air) analyzed at p2 to p12. (B) Image of one of the western blots showing AcH3 expression in the cerebellum isolated from ethanol-exposed pups analyzed at p2 to p12. (C) Quantitative analysis showing that AcH3 protein levels (normalized to β-actin) are lower in the ethanol group compared to the control group between P2 and P10, but not at P12. (D) Quantitative analysis showing that total H3 protein levels (normalized to β-actin) are not changed in the ethanol group compared to the control group. (E) Quantitative analysis showing that the ratio of AcH3K23 compared to total H3 protein levels are lower in the ethanol group compared to the control group between P2 and P12. Three independently exposed liters (n = 3) were analyzed and each was counted as one replicate. Multiple western blots were analyzed for each exposure to minimize the variation between western blots. For P2 and P10, only two independent liters were analyzed therefore no statistic analysis was done on the data for these two time points. (PDF) [file pone.0019351.s002.pdf]
